# Widom Lines in Binary Mixtures of Supercritical Fluids

**DOI:** 10.1038/s41598-017-03334-3

**Published:** 2017-06-08

**Authors:** Muralikrishna Raju, Daniel T. Banuti, Peter C. Ma, Matthias Ihme

**Affiliations:** Department of Mechanical Engineering, Stanford University, Stanford, CA 94305 USA

## Abstract

Recent experiments on pure fluids have identified distinct liquid-like and gas-like regimes even under supercritical conditions. The supercritical liquid-gas transition is marked by maxima in response functions that define a line emanating from the critical point, referred to as Widom line. However, the structure of analogous state transitions in mixtures of supercritical fluids has not been determined, and it is not clear whether a Widom line can be identified for binary mixtures. Here, we present first evidence for the existence of multiple Widom lines in binary mixtures from molecular dynamics simulations. By considering mixtures of noble gases, we show that, depending on the phase behavior, mixtures transition from a liquid-like to a gas-like regime via distinctly different pathways, leading to phase relationships of surprising complexity and variety. Specifically, we show that miscible binary mixtures have behavior analogous to a pure fluid and the supercritical state space is characterized by a single liquid-gas transition. In contrast, immiscible binary mixture undergo a phase separation in which the clusters transition separately at different temperatures, resulting in multiple distinct Widom lines. The presence of this unique transition behavior emphasizes the complexity of the supercritical state to be expected in high-order mixtures of practical relevance.

## Introduction

Supercritical fluids represent an intriguing state of matter, simultaneously exhibiting properties of liquids and gases. The supercritical region is commonly defined as a single-fluid phase beyond the critical point where there exists no physical observable that distinguishes a liquid from a gas. They occur in nature and are considered critical for the origin of life in submarine hydrothermal vents^1^, the discovery of alien life^2^ and the understanding of atmospheric structures on giant planets^3^. In addition, supercritical fluids have practical relevance in industrial applications to cleaning, separation and extraction due to their high density, solubility and transport properties. The utilization of supercritical fluids ranges from carbon capture and storage, biotechnology and food processing^4–7^ to applications in propulsion systems^8^ pertaining to liquid rocket motors, high-pressure gas turbines, and internal combustion engines. Over the past decade, significant progress has been made on the fundamental understanding of properties of supercritical fluids^9–13^. This progress can be largely attributed to advances in experimental methods^14, 15^ and broad commercial use of supercritical technologies. Despite this, important questions concerning the physical micro- and macroscopic behavior of supercritical fluids remain open.

A particular research direction that is leading to new and unexpected insight is the structure of the supercritical state space. The coexistence line of a pure fluid separates liquids from gases; it ends at the critical point beyond which no phase equilibrium is possible. It is widely believed that the supercritical state is homogeneous with no structural and dynamic observable to distinguish between a liquid and a vapor. However, recent experiments have identified regions of distinct liquid or gaseous properties even under supercritical conditions^10, 16^. The supercritical liquid-gas transition occurs across an extension to the coexistence line, marked by almost discontinuous changes in fluid properties. This line was first identified experimentally by Nishikawa and Tanaka^17^ and stands in contrast to the classical presentation of the supercritical state space as a featureless, homogeneous domain. Several concepts are discussed in the literature concerning the demarcation between liquid-like and gas-like supercritical states^10, 16, 17^. Sciortino *et al*.^18^ introduced the definition of a Widom line as the set of states with a maximum correlation length of the fluid^10, 19^, but is often approximated as locus of maximum thermodynamic response functions, as they can be more readily evaluated from thermodynamic quantities. In pure fluids, the maxima of different response functions can be located far from each other^20, 21^. In particular, the specific isobaric heat capacity is a common choice^22–26^. Several researchers suggested that crossing this line is a phase-change-like process, albeit continuous^18, 24, 27^. An alternative supercritical transition was proposed by Fisher and Widom^28^, determined by a transition of the pair-correlation function from an oscillatory to a monotonous behavior, and by Brazhkin *et al*.^29^, characterized by the crossover from an oscillating to a monotonically decaying velocity autocorrelation function^30^. Widom lines have also been observed in magnetic phase transitions^31^ as well as in miscible isotopic mixtures^30^. Previous studies show that impurities shift the location of the Widom line in supercritical CO_2_^32^ and H_2_O^33^.

The Widom line is commonly associated with the thermodynamic transition of a pure fluid, and has been studied for a number of single species, such as CO_2_^17, 32, 34–36^, O_2_^16^, Ar^10, 29^, and H_2_O^37, 38^. However, most practical applications, such as carbon capture or supercritical fluid injection, require the consideration of mixtures of multiple species. As such, there is a fundamental knowledge gap in extending the description of thermodynamic transition states to multicomponent systems. The existence and the range of persistence of the Widom line is of great technological value for fluid mixtures, as it can indicate the pressure and temperature range over which the desired properties can be enhanced. Given the drastic changes in fluid properties across the Widom line, this is immediately relevant for all supercritical systems, and it is of direct importance for developing a new fundamental understanding of the supercritical state space of multicomponent mixtures.

The present paper addresses this issue using molecular dynamics (MD) simulations of binary mixtures as prototypical examples of multicomponent systems. These atomistic-scale investigations contribute significantly to understanding the supercritical state space of mixtures and extract physical details that are not accessible experimentally. It is important to note that even binary mixtures may exhibit very complex mixing behavior^39, 40^. A first theoretical classification of the different mixing phenomena was given by van Konynenburg and Scott^41^. Based on the projection of the critical curves on the *p*-*T* state space and using the van-der-Waals equation of state (EOS), they classified the phase behavior for binary mixtures into six types. Type-I mixtures have a continuous gas-liquid critical line connecting the critical points of the pure components and exhibit complete miscibility of the liquid phases at all temperatures. Usual conditions under which a binary mixture exhibits type-I behavior are that both substances are of similar chemical types and/or their critical properties are comparable in magnitude. Examples are mixtures of argon/krypton, methane/ethane, nitrogen/methane, alkane/alkene or carbon dioxide/methane. Type-II and VI mixtures additionally exhibit liquid-liquid immiscibility at temperatures below the gas-liquid critical line. Type-III mixtures arise in binary mixtures where the region of liquid-liquid immiscibility extends to the gas-liquid critical line making it discontinuous. The disparity in intermolecular forces are particularly significant for the two constituents in this class. Mixtures of neon/krypton, nitrogen/hydrocarbons, water/hydrocarbons or hydrogen/oxygen are typical examples for this phase behavior. Type-IV and V mixtures are characterized by discontinuous gas-liquid critical curves^39^. Type-I and III mixtures can be considered as the two extremes among the various classes. For instance, in studies of polar/nonpolar mixtures, as the dipole moment of the polar constituent is increased, the mixture transforms from type-I to type-II and then to type-III, with types IV and V appearing as intermediate stages, depending on the size and interaction energies of the molecules.

Motivated by their significantly different phase behavior and technological relevance, we investigate binary type-I and III mixtures by considering Ar/Kr and Ne/Kr as respective representative configurations. Interestingly, we discover that thermodynamic transition lines exist in mixtures and – depending on their behavior – the mixtures can exhibit one or multiple Widom lines. We find that current numerical models employing various state equations show significant deficiencies in capturing this observed nonlinear thermodynamic behavior. The MD-simulations in this study were performed using a recently developed ReaxFF reactive force field^42^. We chose the ReaxFF force field because it gives the critical states of Ne, Ar and Kr in good agreement with experimental values. The values of the critical points (*T*_*C*_, *p*_*C*_) from ReaxFF/experiments for Ne, Ar and Kr are (44.4 K, 26.15 atm)/(44.4 K, 27.32 atm), (146.1 K, 47.07 atm)/(150.72 K, 48.63 atm) and (204.69 K, 56.64 atm)/(209.48 K, 55.25 atm), respectively. The development and validation of the force field is provided as supplementary material.

## Results

### Ar/Kr binary mixture

To examine the supercritical state behavior of mixtures with type-I phase behavior, we perform MD-simulations of an equimolar binary Ar/Kr system^43^. Ar and Kr are chemically similar and have comparable critical points. The MD-simulations were performed at temperatures between *T* = 115 K and *T* = 275 K, at increments of 5 K, and for various pressures between *p* = 65 atm and *p* = 140 atm. We found that a temperature interval of 5 K is sufficient to illustrate energetic and structural differences between the liquid-gas phase transition at sub- and supercritical pressures. The simulated isobars cross the liquid and supercritical regions at pressures above the critical pressures, *p*_*C*_, of both mixture components, Ar (48.63 atm) and Kr (55.25 atm).

#### Thermodynamic properties in the supercritical region

**Figure 1:**
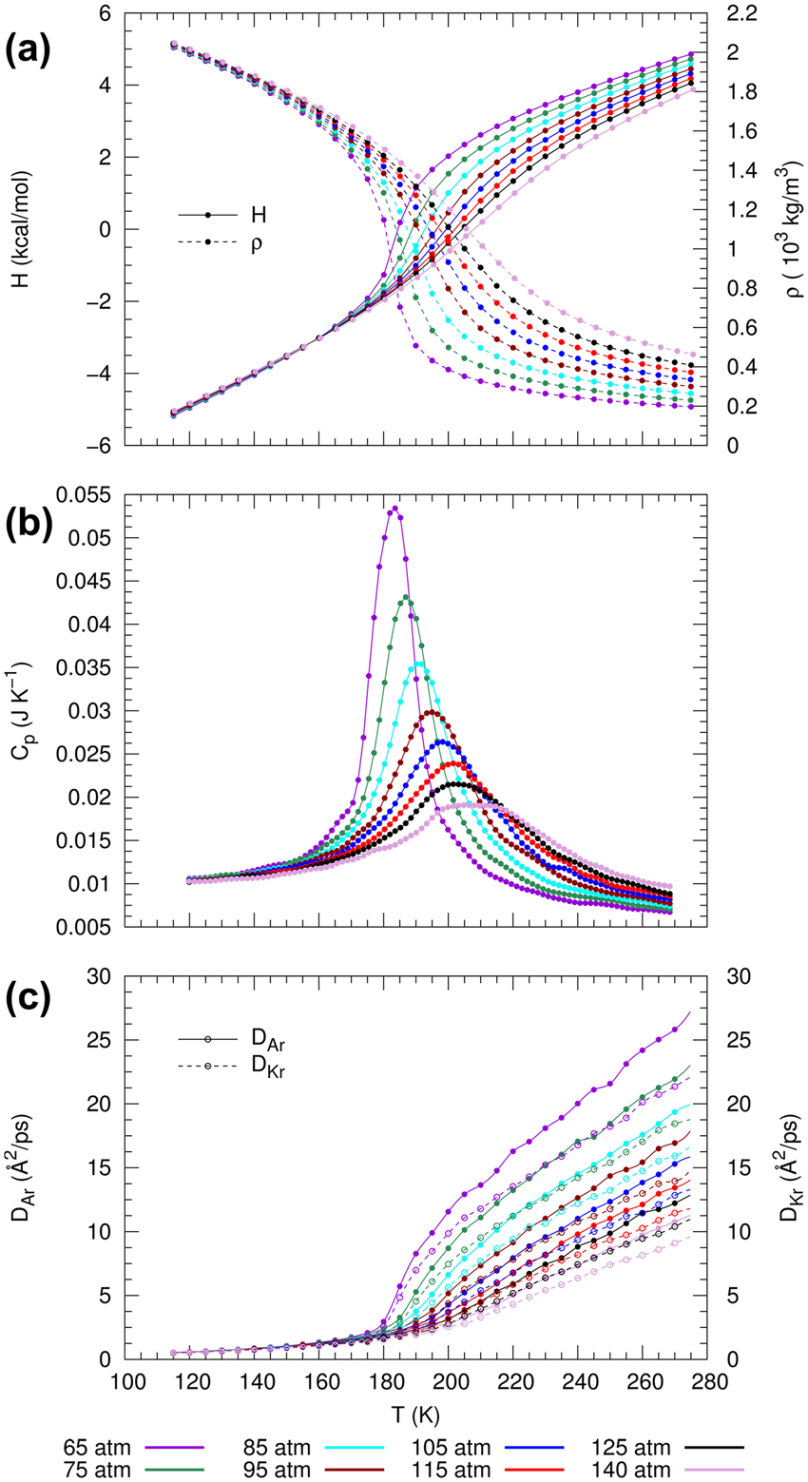
Thermophysical properties and response functions for equimolar Ar/Kr mixture with type-I phase behavior, showing supercritical isobars of (**a**) enthalpy *H* and density *ρ*, (**b**) isobaric heat capacity *C*_*p*_ and (**c**) diffusion coefficients (*D*_Ar_ and *D*_Kr_) as functions of temperature.

Figure 1(a) shows isobars of enthalpy, *H*, and density, *ρ*, for the Ar/Kr mixture. The variation of enthalpy and density with temperature shows a phase transition that occurs over an extended temperature range and is devoid of discontinuous jumps.

To determine the location of the Widom line, we determine the maxima of three thermodynamic response functions, namely the constant-pressure heat capacity, the expansion coefficient, and the isothermal compressibility, respectively:

$${C}_{p}=\frac{1}{N}{(\frac{\partial H}{\partial T})}_{p},\quad {\alpha }_{p}=-\frac{1}{\rho }{(\frac{\partial \rho }{\partial T})}_{p},\quad {\kappa }_{T}=\frac{1}{\rho }{(\frac{\partial \rho }{\partial p})}_{T}.$$

**Figure 2:**
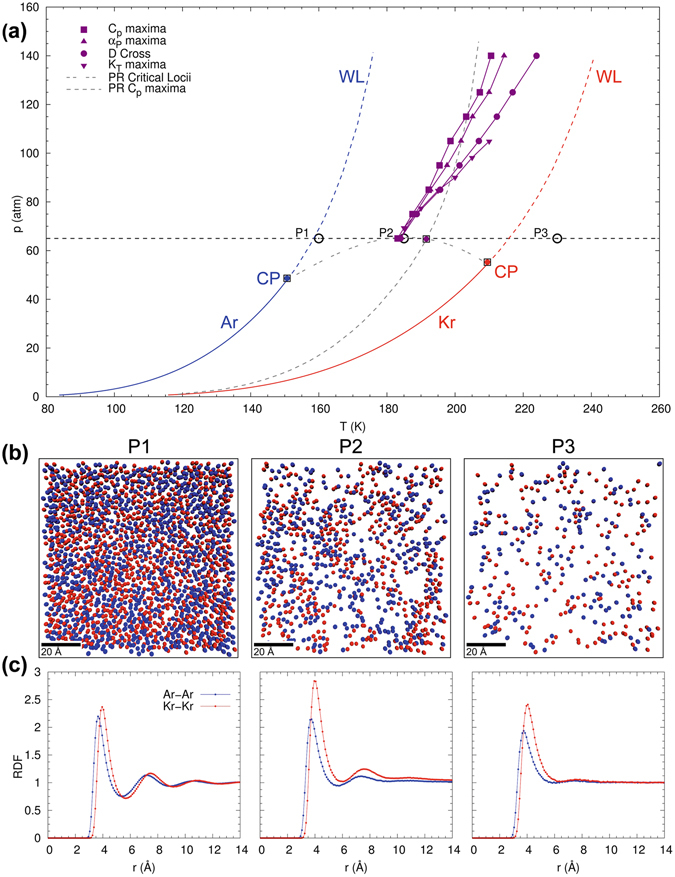
Supercritical state space and molecular structure of equimolar Ar/Kr mixture with type-I phase behavior, showing (**a**) projected *p*-*T* state plane with locations of the maxima of *C*_*p*_, *α*_*p*_, *κ*_*T*_ and diffusion coefficient crossovers as obtained from isobaric MD-simulations; (**b**) MD-snapshots of molecular structure and (**c**) radial distribution function at a supercritical pressure of 65 atm and three different temperatures (160 K, 180 K, and 230 K; from left to right).

The computed behavior of *C*_*p*_ as a function of temperature for different pressures is shown in Fig. 1(b). Despite the fact that the pressure exceeds *p*_*C*_ of the individual components, the peak of the heat capacity is well defined for pressures far greater than the liquid-vapor critical points (LVCPs). This peak decays with increasing pressure as expected from the theoretical understanding of the Widom line and can be observed even up to a value of approximately 3*p*_*C*_ of Kr. This persistence of a well-defined maximum at pressures far above the LVCPs is also observed for *α*_*p*_ and *κ*_*T*_ (not shown). The location for the maxima of *C*_*p*_, *α*_*p*_ and *κ*_*T*_ in the projected *p*-*T* state plane are illustrated in Fig. 2(a). For reference, coexistence lines and Widom lines for the pure species of Ar and Kr from the NIST-database (http://webbook.nist.gov/chemistry/fluid) are shown in blue and red, respectively, and purple lines with symbols correspond to computational results from isobaric MD calculations. We also included results from the Peng-Robinson (PR) state equation and vapor-liquid equilibrium computations to infer the Widom line and gas-liquid critical curve, respectively, in order to facilitate comparisons with commonly employed macroscopic model approaches.

#### Transport properties in the supercritical region

We now consider the dynamic properties in the supercritical region. The self-diffusion coefficients of Ar and Kr, *D*_Ar_ and *D*_Kr_, in the binary mixture are calculated from the MD-simulations as shown in Fig. 1(c). At conditions near the critical pressure, the curves show a drastic change of slope close to the phase transition. We can observe that changes in slope of *D*_Ar_ and *D*_Kr_ coincide, indicating that Ar and Kr undergo a simultaneous and continuous transition from a liquid-like to a gas-like phase. At higher pressures the curves progressively become linear, which is also observed in the behavior of enthalpy and density (cf. Fig. 1(a)).

At conditions close to the critical pressure, it is possible to distinguish between liquid-like and gas-like phases by fitting the diffusion coefficient to the Arrhenius formula^44^, $D={D}_{0}\,\exp \,\{-{E}_{A}/({k}_{B}T)\}$, where *E*_*A*_ is the activation energy and *k*_*B*_ is the Boltzmann constant. Activated processes with different activation energies determine the diffusion in the liquid-like and gas-like regions. For the simulated pressures a good fit for diffusion is realized by using different values for *E*_*A*_ in the liquid-like (low *T*) and gas-like (high *T*) regions. From the behavior of the self-diffusion coefficients for Ar and Kr, we evaluate the location of the Widom line as the point at which the curvature changes. To precisely locate the inflection point we calculate the numerical derivatives of the diffusion curves.

Figure 2(a) shows the locus of the Widom line in the projected *p*-*T* state-diagram obtained from maxima of *C*_*p*_, *α*_*p*_, and *κ*_*T*_ and the diffusion crossover point (DCP) as obtained from isobaric MD simulations. We note that the loci of the response functions coincide perfectly up to a pressure of 80 atm, which is approximately 25 atm above the critical pressure of the least volatile component in this mixture. Above this condition, the loci for *C*_*p*_ and *α*_*p*_ continue to coincide, while those for *κ*_*T*_ and DCP deviate concurrently. For comparison, we also plot the maxima of *C*_*p*_ obtained from the Peng-Robinson equation of state using mixing rules (see Supplementary material). The LVCP and Widom line predicted by the PR-EOS are shifted slightly to higher temperatures. Excluding this slight difference in temperatures, results from the macroscopic state relation and MD-simulations exhibit remarkably similar variation with pressure. This in turn suggests that the PR-EOS can describe supercritical mixtures that are representative of type-I phase behavior.

Interestingly, we note that the supercritical *p*-*T* state space of this binary Ar/Kr mixture exhibits similar behavior to that of a single component fluid (see Supplementary material). Both the Ar/Kr mixture and pure Ar exhibit a single set of Widom lines that emanate from the LVCP. We investigate the structure of the Ar/Kr mixture to understand the similarity to the pure-fluid behavior. The MD-snapshots at temperatures below, during and after the phase-transition at 65 atm are shown in Fig. 2(b). From this, it can be observed that the mixture is homogeneous and devoid of any phase separations. The corresponding Ar/Ar and Kr/Kr radial distribution functions (RDF) are shown in Fig. 2(c) and we immediately observe that Ar and Kr have perfectly similar RDFs across the phase transition. The MD-snapshots and similarity of the Ar/Ar and Kr/Kr RDFs suggest that the mixture is homogeneous and that Ar and Kr transform simultaneously from a liquid-like to a gas-like phase. This is also in agreement with the concurrent change in slope of *D*_Ar_ and *D*_Kr_ in Fig. 1(c). Thus, the homogeneity and miscibility of the mixture gives rise to the pure fluid-like behavior of Ar/Kr mixtures.

### Ne/Kr binary mixture

Next, we examine a Ne/Kr mixture, which exhibits type-III phase behavior^45^. Because of the difference in critical properties between both components, these mixtures are classified by the absence of a continuous gas-liquid critical curve and liquid-liquid immiscibility. To examine the phase transition and the Widom line, MD-simulations of an equimolar mixture were performed at temperatures between *T* = 25 K and *T* = 275 K in increments of 5 K and for pressures between *p* = 35 atm and *p* = 140 atm.

#### Thermodynamic properties in the supercritical region

**Figure 3:**
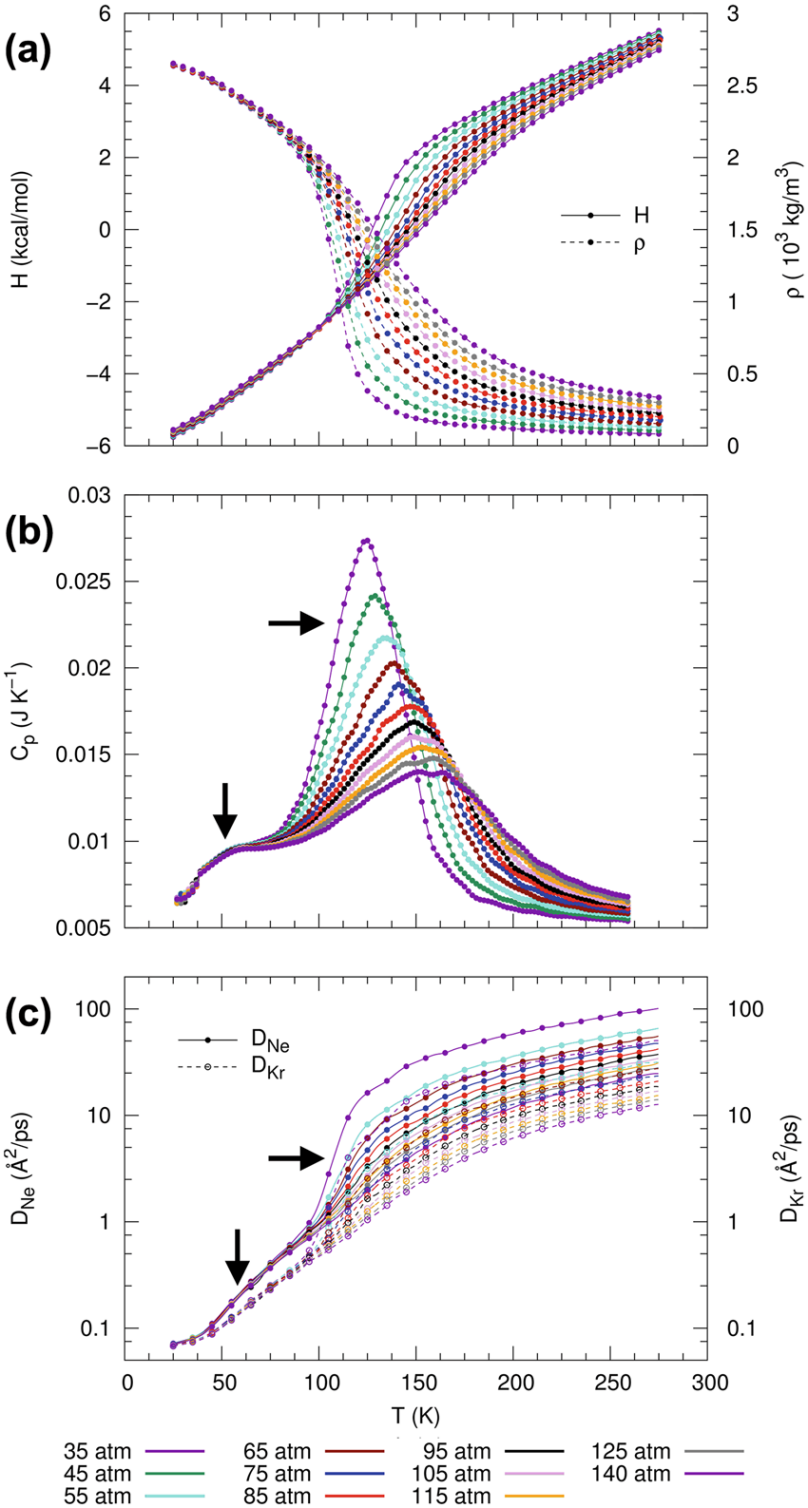
Thermophysical properties and response functions for equimolar Ne/Kr mixture with type-III phase behavior, showing (**a**) supercritical isobars of enthalpy *H* and density *ρ*, (**b**) isobaric heat capacity *C*_*p*_ as a function of temperature, and (**c**) diffusion coefficients (*D*_Ne_ and *D*_Kr_) as obtained along the simulated isobars.

Isobars of enthalpy, heat capacity, and diffusivities as a continuous function of temperature and for different pressures are shown in Fig. 3. Interestingly, a drastically different phase-transition behavior is observed for this type-III Nr/Kr mixture as compared to the type-I Ar/Kr mixture. In particular, it can be seen from Fig. 3(a) that the isobars of enthalpy and density show the presence of two distinct phase transitions at approximately 50 K and 130 K. The presence of two transitions is also evident by the behavior of *C*_*p*_, exhibiting two separate peaks as shown in Fig. 3(b). The magnitude of the *C*_*p*_ peaks decreases with increasing pressure and persists even up to pressures above 140 atm. The observation of two phase transitions in the Ne/Kr mixture indicates the occurrence of Widom lines at two distinct locations in the projected *p*-*T* state diagram, thereby demonstrating the separated phase transition of each component for this type-III mixture.

#### Transport properties in the supercritical region

In Fig. 3(c), we report the diffusion coefficients of Ne and Kr as calculated from the MD-trajectories. We can observe that changes in *D*_Ne_ and *D*_Kr_ initiate at different temperatures, providing further evidence that Ne and Kr transition separately from a liquid-like to a gas-like regime. This is consistent with the behavior of enthalpy and density. Thus, analysis of both thermodynamic properties and dynamic behavior of the Ne/Kr mixture confirms the presence of two distinct phase transitions, resulting in two sets of Widom lines. This is a new and unexpected behavior in the supercritical state space, where we observe two consecutive changes in fluid properties, otherwise unobserved in pure fluids and binary Ar/Kr mixtures with type-I phase behavior.

**Figure 4:**
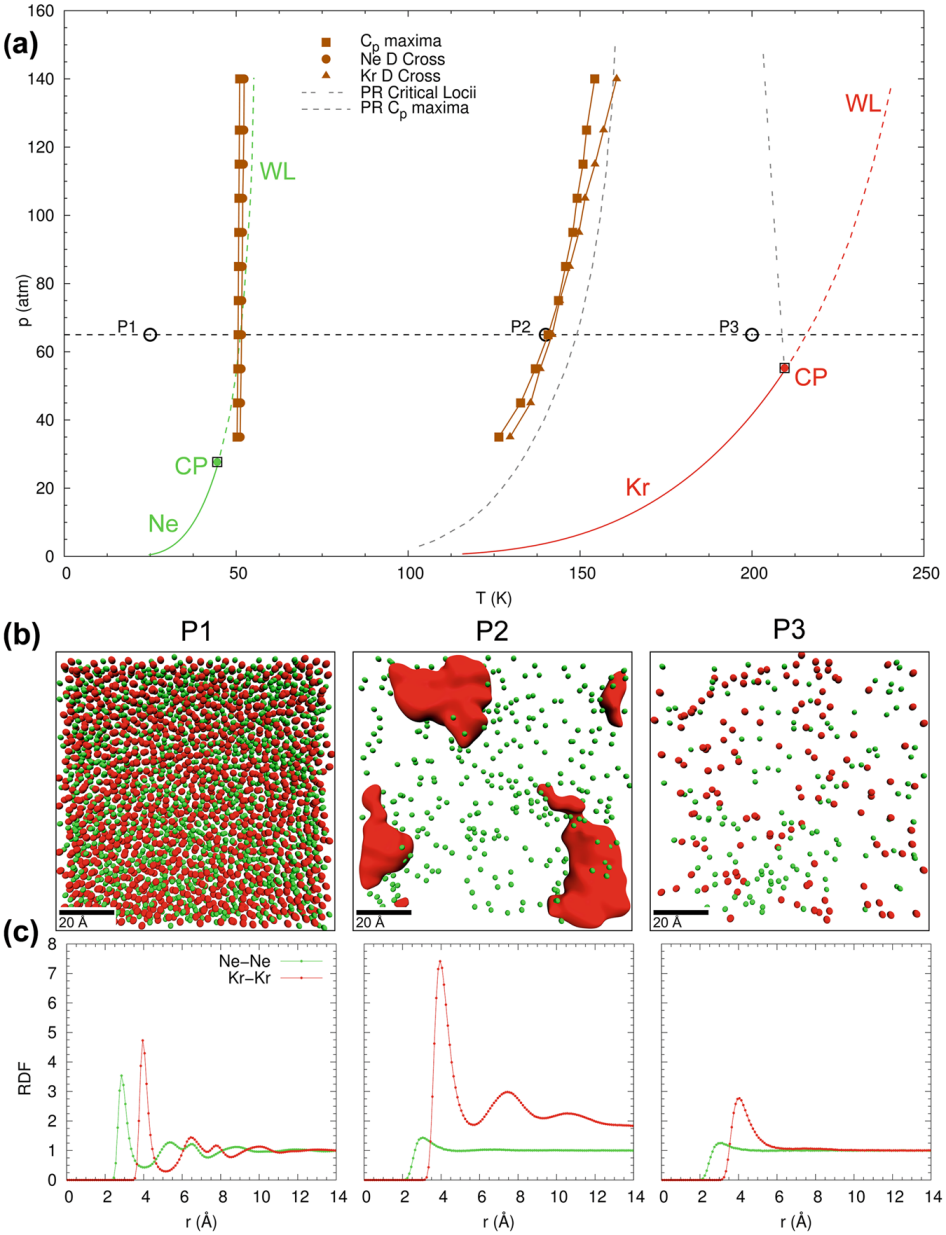
Supercritical state space and molecular structure of equimolar Ne/Kr mixture with type-III phase behavior, showing (**a**) projected *p*-*T* state plane with locations of the maxima of *C*_*p*_, *α*_*p*_, *κ*_*T*_ and diffusion coefficient crossovers as obtained from isobaric MD-simulations; (**b**) MD-snapshots of molecular structure and (**c**) radial distribution function at a supercritical pressure of 65 atm and three different temperatures (30 K, 140 K, and 200 K; from left to right).

We now evaluate the loci of the two distinct Widom lines in the projected *p*-*T* state-diagram, as obtained from *C*_*p*_ maxima and DCP evaluated along isobars. These results are presented in Fig. 4(a), showing that the Ne/Kr mixture exhibits a completely different phase transition behavior as compared to the Ar/Kr mixture (cf. Fig. 2). It is important to appreciate that the two distinct phase transitions in this mixture are not identical to the Widom lines of either pure Ne or pure Kr. This shows that even though the mixture components are immiscible, their interactions at the phase boundaries affect the transition point of the mixture. The two distinct phase transitions, as indicated by the thermodynamic and dynamic markers, give rise to two sets of Widom lines in the state diagram, originating at circa 50 K and 130 K. Around 50 K, the *C*_*p*_ and DCP lines coincide perfectly up to the highest simulated pressure of 140 atm. Around 130 K, the Widom lines coincide up to 100 atm and then begin to deviate. In comparison, the *C*_*p*_ maxima obtained from PR-EOS predict only a single Widom line for the Ne/Kr mixture at *T* ~ 140 K. Although the Peng-Robinson state equation (gray dashed line in Fig. 4(a)) predicts a Widom line whose location is in qualitative agreement with the Widom line given by the MD-simulation at *T* ~ 135 K, a detailed analysis showed that the structure of the *C*_*p*_ profile is not captured. Furthermore, the cubic state equation completely misses the Widom line at *T* ~ 50 K. This suggests the need for developing new models for the thermodynamic description of immiscible mixtures that are characterized by type-III behavior. This is relevant for several technical applications, involving mixtures that contain components with well separated critical points.

MD-snapshots (Fig. 4(b)) and structural analysis (Fig. 4(c)) provide further insight into the origin of the two distinct phase transitions of the Ne/Kr mixture. Specifically, the MD-snapshots show that the mixture is not homogeneous: the components phase-separate and form isolated clusters of pure Ne or pure Kr. Radial distribution functions for Ne/Ne and Kr/Kr, respectively, at various temperatures along the isobar at 65 atm show that Ne transitions to a gas-like regime at an appreciably lower temperature than Kr. The individual components phase-separate in two distinct steps: (i) Ne transitions to a gas-like regime at *T* ~ 50 K, followed by (ii) Kr clusters, which transition to a gas-like regime at *T* ~ 120 K, with the exact transition temperature varying with pressure. The structural analysis reveals that the immiscibility of Ne and Kr gives rise to two distinct phase transitions and thereby two Widom lines. The mixture becomes homogeneous at higher temperatures after both Ne and Kr transition to a gas-like regime.

We note that the attractive interactions of Ne/Ne are weaker than Kr/Kr. As a result, changes in fluid properties associated with the phase transition of Ne to a gas-like regime at *T* ~ 50 K are smaller in magnitude compared to the phase transition at *T* ~ 130 K involving Kr. This is, for instance, evident in the smaller *C*_*p*_ peak corresponding to the phase transition of Ne. To further demonstrate the significance of these distinct phase transitions, we extend this analysis by creating a model noble-gas mixture in which the two individual components have strong self-interactions. We label the two atoms as Ne′ and Ar′, since their force field parameters were developed from regular Ne and Ar parameters by making their self-interactions more attractive. In this model system, Ne′ and Ar′ have critical points (30.1 K, 41.35 atm) and (269.1 K, 111.52 atm), respectively. The force field parameters are provided as supplementary material. Results from this analysis are discussed in the next section. In this context it is important to note that mixtures transform from type-I to type-III phase behavior with increasing dipole moment of the polar constituent in polar/non-polar mixtures.

### Model noble gas mixture with strong self-interaction

**Figure 5:**
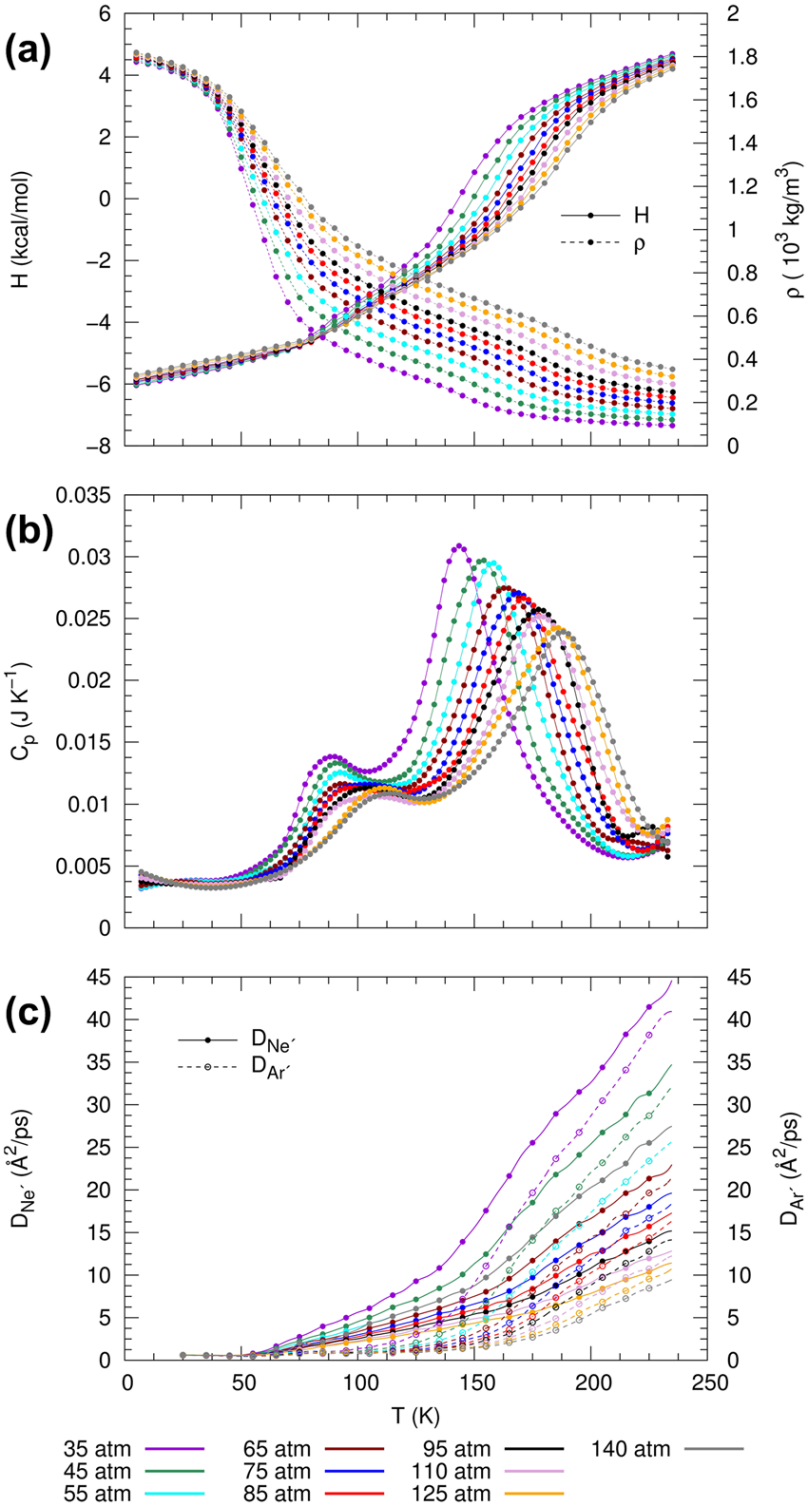
Thermophysical properties and response functions for equimolar Ne′/Ar′ mixture with strong self-interaction, showing (**a**) supercritical isobars of enthalpy *H* and density *ρ*, (**b**) isobaric heat capacity *C*_*p*_ as a function of temperature, and (**c**) diffusion coefficients (*D*_Ne′_ and *D*_Kr′_) as obtained along the simulated isobars.

To further delineate the two distinct phase transitions and highlight the importance of self-interaction energies, we perform MD-simulations of the binary model system Ne′/Ar′. The isobars of enthalpy and density for this system are shown in Fig. 5(a), from which we can observe two distinct phase transitions. The presence of these pronounced phase transitions can also be expected in realistic fluid mixtures where the intermolecular interactions are not just van-der-Waals forces as in the noble-gas mixtures considered here. The behavior of the heat capacity and the diffusivity of the model system also shows the presence of two distinct phase transitions (cf. Fig. 5(b,c), respectively). We can immediately observe that the first peak in *C*_*p*_ at low temperatures is significantly more pronounced compared to that of the Ne/Kr system. The analysis of the expansion coefficient (not presented) showed that both peaks have comparable magnitudes, indicating two successive and drastic changes in volume. Furthermore, the inflection points in the diffusivity of the two atoms show an appreciable temperature separation. These distinct changes in thermodynamics and dynamic properties at two separate locations in the projected *p*-*T* state space can be critical for further development and deployment of supercritical mixtures.

**Figure 6:**
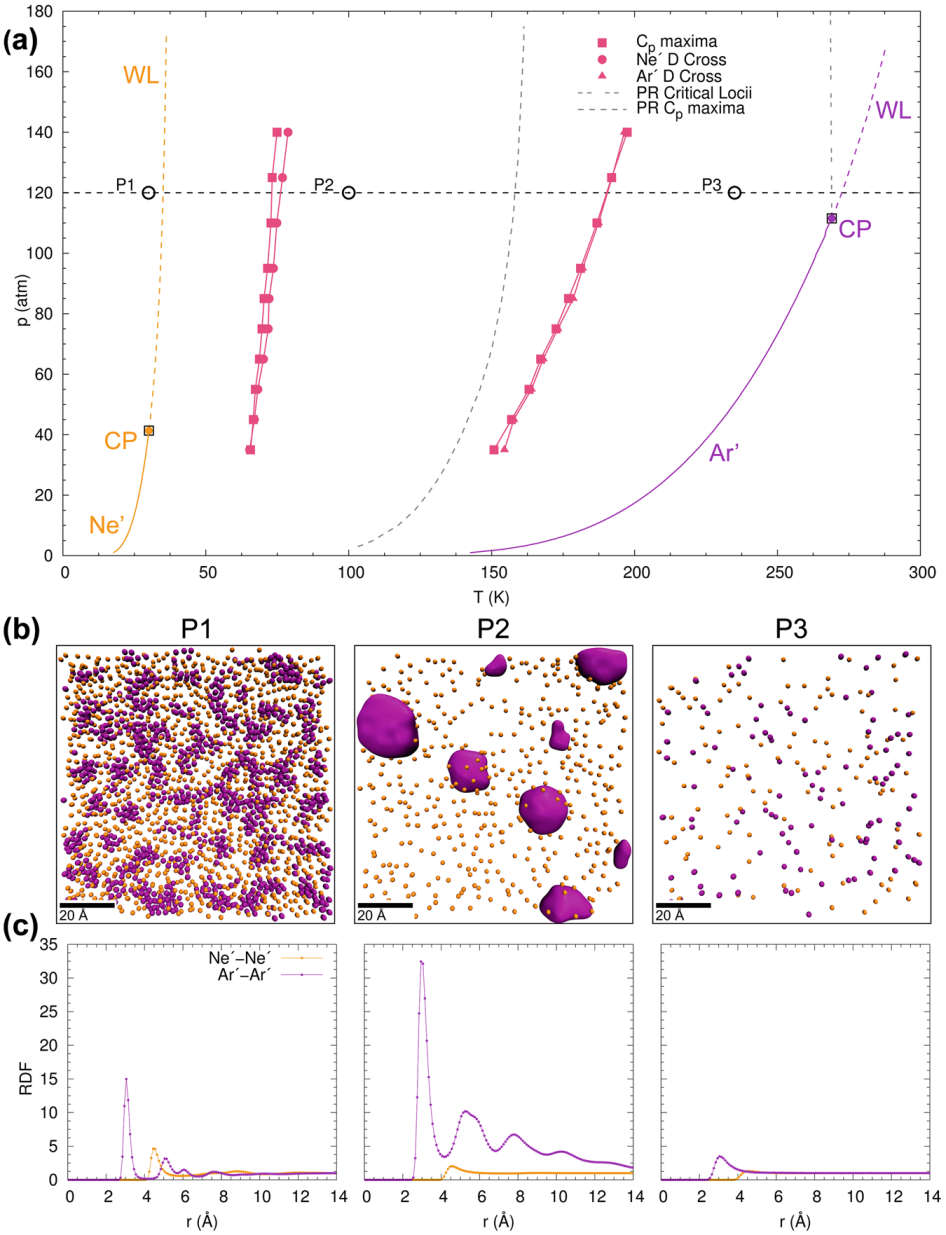
Supercritical state space and molecular structure of equimolar Ne′/Ar′ mixture with strong self-interaction, showing (**a**) projected *p*-*T* state plane with locations of the maxima of *C*_*p*_, *α*_*p*_, *κ*_*T*_ and diffusion coefficient crossovers as obtained from isobaric MD-simulations; (**b**) MD-snapshots of molecular structure and (**c**) radial distribution function at a supercritical pressure of 120 atm and three different temperatures (30 K, 100 K, and 235 K; from left to right).

Figure 6(a) shows two sets of Widom lines observed in the model Ne′/Ar′ mixture as obtained from isobaric MD simulations. The Widom lines are anchored at *T* ~ 65 K and *T* ~ 175 K. As observed in the Ne/Kr-mixture, the *C*_*p*_ and DCP loci coincide at lower pressures and deviate with increasing pressure. The MD-snapshots and corresponding RDFs of the model mixture at 30 K, 100 K and 235 K are shown in Fig. 6(b,c), respectively. The model mixture exhibits a phase-transition behavior similar to the Ne/Kr mixture. The individual components phase-separate and transition separately from a liquid-like to a gas-like state. We can observe that at 100 K, Ne′ has transitioned to a gas-like state while the RDF for Ar′ indicates clustering with a liquid-like behavior. After Ar′ transitions to a gas-like state, the mixture becomes homogeneous. This implies that the phase transition occurs in two separate steps, resulting in two sets of Widom lines akin to the binary Ne/Kr mixture. Owing to the strong self-interaction, both phase transitions are clearly visible in the behavior of the thermodynamic and dynamic markers.

## Discussion

We examine the thermodynamic and dynamic properties of binary Ar/Kr and Ne/Kr mixtures in the supercritical region using MD-simulations. For the first time, we demonstrate that binary mixtures undergo drastic changes in fluid properties even up to pressures in excess of 3*p*_*C*_ of the more volatile component. However, with increasing pressure, the transition from a liquid-like to a gas-like state becomes progressively continuous with smoother changes in the fluid properties, thereby approaching a “true” supercritical phase. Our simulations suggest that technical applications in this operating range have to recognize and adequately represent these changes in fluid properties.

To extract the trajectories of the Widom lines in the projected *p*-*T* state space, we evaluate the maxima of *C*_*p*_, *α*_*p*_ and *κ*_*T*_ in the region above the critical points of the individual mixture components. Depending on the phase behavior of the binary mixture, we observe one or two sets of Widom lines. In particular, the homogeneous Ar/Kr binary mixture with miscible type-I mixing behavior exhibits a single set of Widom lines, having a phase-transition behavior similar to a pure fluid. This behavior can be expected in mixtures, where the individual molecules have comparable critical points. In contrast, the binary Ne/Kr mixture, exhibiting immiscible type-III behavior, shows separate phase transitions, giving rise to two Widom lines that are distinctly different from those of each pure component. While previous investigations exclusively focused on the analysis of pure mixtures, the present investigation reveals a new and unexpected behavior with immediate relevance to fundamental understanding and technical applications. For instance, this behavior can be important for describing fuel-injection systems at transcritical conditions, where the critical point of the hydrocarbon fuel is well separated from the oxidizer.

Results from this investigation expand upon our understanding of the supercritical state space from recent experimental^9, 10^ and theoretical studies^3, 44^ of pure fluids to binary mixtures. The supercritical region in mixtures can therefore no longer be considered as an indistinguishable homogeneous fluid phase, but rather as a complex state space where components transition from a liquid-like to a gas-like state either simultaneously or separately, giving rise to a multifeatured state space. In addition, we show that current numerical models employed in macroscopic simulations fail to adequately capture phase transitions of immiscible mixtures, emphasizing the need for formulating new models to describe supercritical fluid mixtures accurately.

Motivated by these theoretical findings, experimental investigations are warranted to substantiate these results and extend the study to mixtures containing molecules that exhibit more complex intermolecular interactions, such as H_2_O, CO_2_ and hydrocarbons. Concerning experimental evidence, recent reports on Widom lines in pure fluid phases^9, 10^ are encouraging.

## Electronic supplementary material

Supplementary Material

ReaxFF Force Field for Ne/Ar model system

ReaxFF Force Field for Ne/Ar/Kr system
