# Supplementary material for: Widom Lines in Binary Mixtures of Supercritical Fluids
Source: Sci Rep. 2017 Jun 8;7:3027. doi: 10.1038/s41598-017-03334-3 (PMC5465206; doi:10.1038/s41598-017-03334-3)
Supplement: Supplementary file 1 — Supplementary Material [file 41598_2017_3334_MOESM1_ESM.pdf]

Supplementary Material: Widom Lines in Binary Mixtures of Supercritical Fluids

Muralikrishna Raju[1], Daniel T. Banuti[1], Peter C. Ma[1], and Matthias Ihme[1,*]

[1]Department of Mechanical Engineering, Stanford University, Stanford, CA 94305
[*]mihme@stanford.edu

Methods

Molecular dynamics simulation: ReaxFF molecular dynamics (MD) simulations were performed using the LAMMPS package[1] in a periodic cubic box containing 25,600 atoms in the canonical N-p-T (constant number of atoms (N), constant pressure (p) and constant temperature (T)) ensemble at different temperatures and pressures. For each pressure, the system was first energy-minimized with convergence criterion of 0.1 kcal/Å. The system was then equilibrated for 62.5 ps and the system energy and other properties were averaged for the following 62.5 ps of the production run. The simulations were run with a time step of 0.25 fs using the Nose-Hoover thermostat with a coupling time constant of 10 fs and Nose-Hoover barostat with a coupling time constant of 100 fs to control the temperature and pressure of the system, respectively. The simulations were performed for pressures ranging up to $3p_C$ (critical pressure) of the mixture component with the higher p_C. The self-diffusion coefficient was obtained from the mean-square displacement through Einstein's relation[2]. For the calculation of the diffusion coefficient, we exclude the first and the last 6.25 ps from the linear fit of the mean square displacement.

Force field development and validation: The Ne/Ar/Kr force field was developed by training the van der Waals parameters in the ReaxFF reactive force field[3] against experimental Ne, Ar and Kr dimer potential energy curves[4,5]. To validate the developed ReaxFF force field we compare the enthalpy obtained from isobaric MD-simulations with experimental enthalpy curves obtained from the National Institute of Standards and Technology (NIST Chemistry WebBook, http://webbook.nist.gov/chemistry/fluid). These MD-simulations were performed at both sub- and supercritical pressures of the pure species.

Figure S1(a) show MD-simulation results for pure Ar, comparing computed Widom lines against results from the NIST-database (as determined from the maxima of the isobaric heat capacity). To facilitate comparisons with the binary mixtures, MD-snapshots and RDFs at three temperatures crossing the liquid-like, transitional, and gas-like state are illustrated in Figs. S1(b, c).

Figure S2 shows comparisons of enthalpy of pure Ar obtained from MD-simulations against experiments at pressures of 35, 65, 144 and 450 atm. We can observe that there is good agreement between the MD-calculations and measurements for all simulated pressures. Comparisons of MD-simulations and experiments for enthalpies of Kr and Ne are illustrated in Fig. S3, showing that the MD-results and experimental enthalpy curves coincide over the entire temperature range. The MD-simulations capture the phase transition temperature in good agreement with experiments.

Peng-Robinson state equation and vapor-liquid equilibrium: To provide a comparison of MD-calculations with macroscopic thermodynamic state evaluations, we consider the Peng-Robinson state equation[6] for its acceptable accuracy and common utilization in technical applications[7]. In this cubic state equation, the two model parameters relating to the intermolecular interaction and volume are functions of critical properties and binary interaction parameters, and are evaluated using van-der-Waals mixing rule[8].

To compute the critical points for the binary mixture, we follow the procedure by Heidemann and Khalil[9]. This method relies on the Taylor-expansion of the Helmholtz free energy, and evaluating the composition by equating the quadratic and cubic expansion term to zero. A nested Newton iteration is employed to determine the temperature and volume at the critical point, from which the corresponding pressure is obtained using the Peng-Robinson EOS.

The criterion for the vapor-liquid equilibrium at a given pressure and temperature is that the partial Gibbs energy of each component in the mixture is equal for the vapor and liquid phases. This is equivalent to equating the partial fugacity of each phase, which is here used to calculate the vapor-liquid equilibrium.

References

1. Plimpton, S. Fast parallel algorithms for short-range molecular-dynamics. *Journal of Computational Physics* **117**, 1–19 (1995).

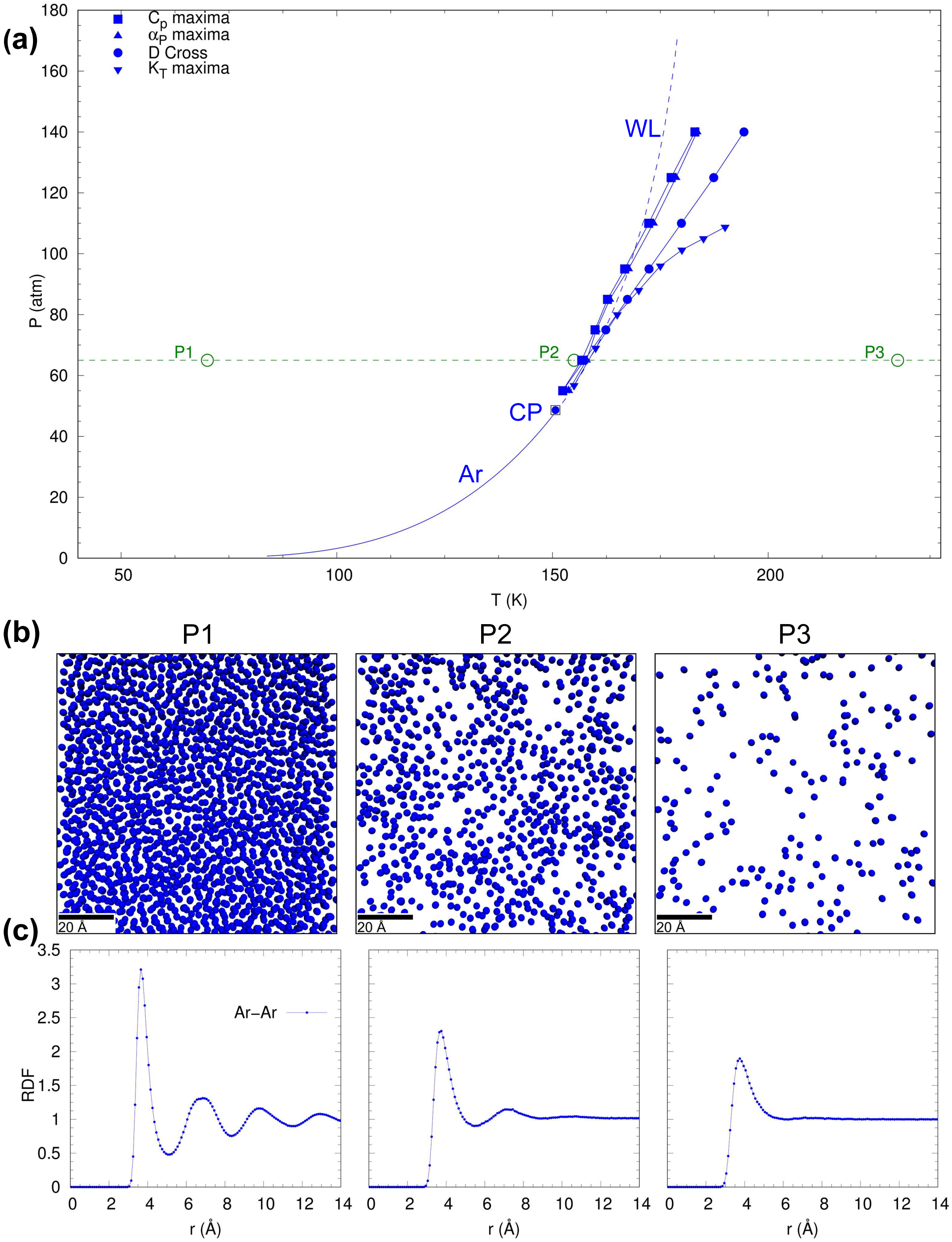

Figure S1. Supercritical state space and molecular structure of pure Ar, showing (a) projected p-T state plane with locations of the maxima of C_p, α_p, κ_T and diffusion coefficient crossovers as obtained from isobaric MD-simulations; (b) MD-snapshots of molecular structure and (c) radial distribution function at a supercritical pressure of 65 atm and three different temperatures (75 K, 155 K, and 230 K; from left to right).

2. Frenkel, D. & Smit, B. *Understanding Molecular Simulation* (Academic Press, 2001), 2nd edn.

3. van Duin, A. C. T., Dasgupta, S., Lorant, F. & Goddard, W. A. ReaxFF: A reactive force field for hydrocarbons. *Journal of Physical Chemistry A* **105**, 9396–9409 (2001).

4. Ogilvie, J. F. & Wang, F. Y. H. Potential-energy functions of diatomic molecules of the noble gases. I. Like nuclear species.

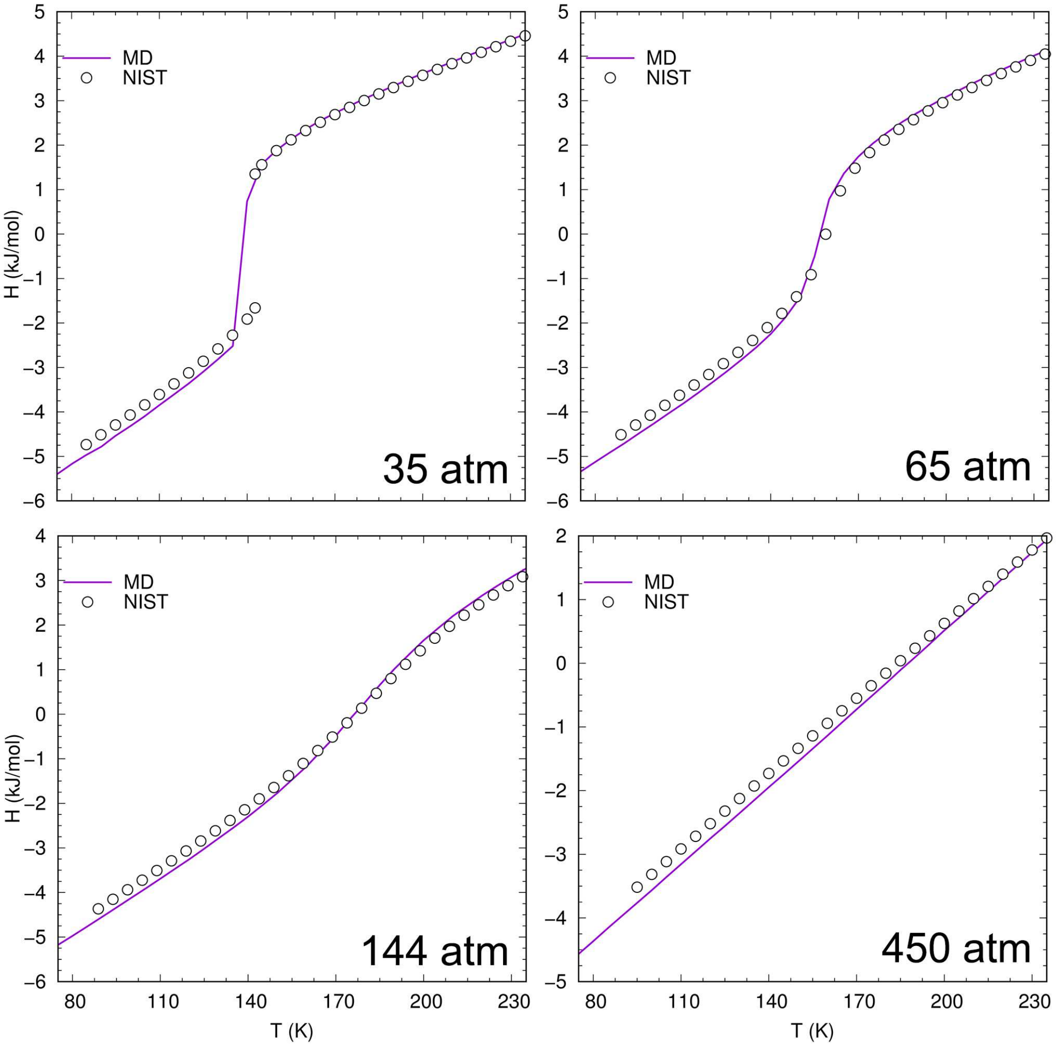

Figure S2. Comparison of enthalpy of pure Ar at different pressures obtained from MD simulations and experiments (NIST).

Journal of Molecular Structure **273**, 277–290 (1992).

5. Ogilvie, J. F. & Wang, F. Y. H. Potential-energy functions of diatomic molecules of the noble gases. II. Unlike nuclear species. *Journal of Molecular Structure* **291**, 313–322 (1993).
6. Peng, D.-Y. & Robinson, D. B. A new two-constant equation of state. *Industrial & Engineering Chemistry Fundamentals* **15**, 59–64 (1976).
7. Harstad, K. G., Miller, R. S. & Bellan, J. Efficient high-pressure state equations. *AIChE Journal* **43**, 1605–1609 (1997).
8. Poling, B. E., Prausnitz, J. M. & O'Connell, J. P. *The Properties of Gases and Liquids* (McGraw-Hill, 2000).
9. Heidemann, R. A. & Khalil, A. M. The calculation of critical points. *AIChE Journal* **26**, 769–779 (1980).

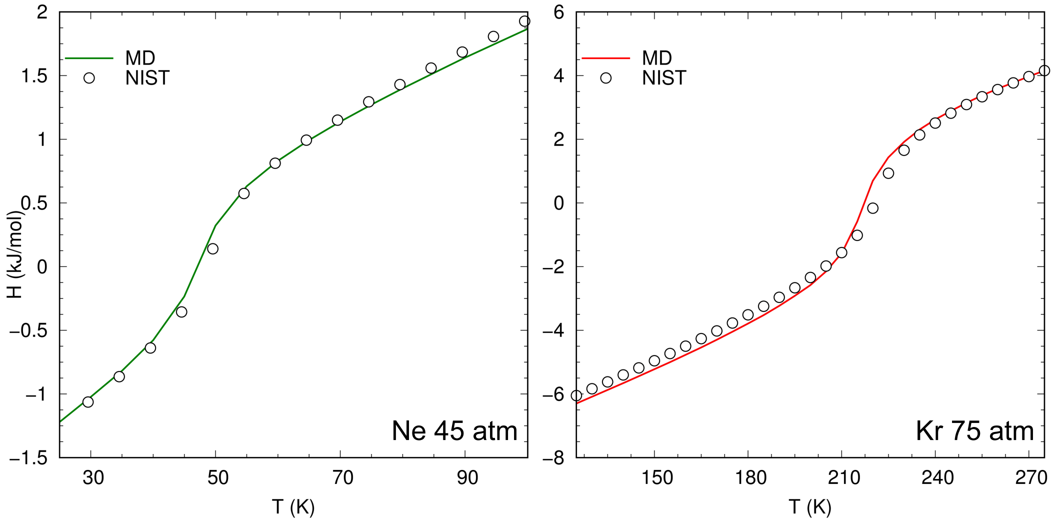

Figure S3. Comparison of enthalpy of pure Ne and Kr obtained from MD-simulations and experiments (NIST).
